# Imaging sequence for joint myocardial T_1_ mapping and fat/water separation

**DOI:** 10.1002/mrm.27390

**Published:** 2018-07-29

**Authors:** Maryam Nezafat, Shiro Nakamori, Tamer A. Basha, Ahmed S. Fahmy, Thomas Hauser, René M. Botnar

**Affiliations:** ^1^ Division of Imaging Sciences and Biomedical Engineering King's College London London United Kingdom; ^2^ Department of Medicine Beth Israel Deaconess Medical Center and Harvard Medical School Boston Massachusetts; ^3^ Biomedical Engineering Department Cairo University Giza Egypt; ^4^ Pontificia Universidad Católica de Chile, Escuela de Ingeniería Santiago Chile

**Keywords:** cardiac magnetic resonance imaging, Dixon, myocardial tissue characterization, T_1_ mapping

## Abstract

**Purpose:**

To develop and evaluate an imaging sequence to simultaneously quantify the epicardial fat volume and myocardial T_1_ relaxation time.

**Methods:**

We introduced a novel simultaneous myocardial T_1_ mapping and fat/water separation sequence (joint T_1_‐fat/water separation). Dixon reconstruction is performed on a dual‐echo data set to generate water/fat images. T_1_ maps are computed using the water images, whereas the epicardial fat volume is calculated from the fat images. A phantom experiment using vials with different T_1_/T_2_ values and a bottle of oil was performed. Additional phantom experiment using vials of mixed fat/water was performed to show the potential of this sequence to mitigate the effect of intravoxel fat on estimated T_1_ maps. In vivo evaluation was performed in 17 subjects. Epicardial fat volume, native myocardial T_1_ measurements and precision were compared among slice‐interleaved T_1_ mapping, Dixon, and the proposed sequence.

**Results:**

In the first phantom, the proposed sequence separated oil from water vials and there were no differences in T_1_ of the fat‐free vials (*P* = .1). In the second phantom, the T_1_ error decreased from 22%, 36%, 57%, and 73% to 8%, 9%, 16%, and 26%, respectively. In vivo there was no difference between myocardial T_1_ values (1067 ± 17 ms versus 1077 ± 24 ms, *P* = .6). The epicardial fat volume was similar for both sequences (54.3 ± 33 cm^3^ versus 52.4 ± 32 cm^3^, *P* = .8).

**Conclusion:**

The proposed sequence provides simultaneous quantification of native myocardial T_1_ and epicardial fat volume. This will eliminate the need for an additional sequence in the cardiac imaging protocol if both measurements are clinically indicated.

## INTRODUCTION

The quantification of the longitudinal relaxation time of the myocardium with MR T_1_ mapping techniques has become an important and reliable biomarker for a number of cardiac diseases such as diffuse myocardial fibrosis.^1^, ^2^, ^3^ The voxel‐wise measurement of the myocardial T_1_ time can provide unique tissue characterization that is not feasible with other imaging modalities. Several relaxometry sequences are available that can obtain the myocardial T_1_ maps, including the modified Look‐Locker inversion recovery (MOLLI),^4^, ^5^ shortened modified Look‐Locker inversion recovery (ShMOLLI),^6^ saturation recovery single‐shot acquisition,^7^ saturation pulse‐prepared heart‐rate‐independent inversion recovery,^8^ and slice‐interleaved T_1_ (STONE) sequence.^9^ All T_1_ relaxation‐time mapping techniques consist of (1) manipulating the longitudinal magnetization, (2) sampling the recovery curve by acquiring multiple images with different T_1_ weighting, and (3) curve fitting of the theoretical inversion/saturation recovery signal to the measured image intensities. The MOLLI sequence is the most commonly used technique to quantify the T_1_ of the myocardium.^10^ One disadvantage of the MOLLI technique is the underestimation of the calculated T_1_ relaxation time.^10^ The reduced accuracy is a result of sensitivity to B_1_ inhomogeneity, off‐resonance (B_0_), heart rate, imperfection of the inversion pulse, and use of an imperfect Look‐Locker correction.^11^, ^12^, ^13^ The STONE T_1_ mapping sequence was proposed to improve the accuracy of T_1_ measurements compared with the well‐established MOLLI sequence while maintaining the same precision and reproducibility. With this technique, multiple images are acquired after a nonselective adiabatic inversion pulse for all slices but with different inversion time (time between the end of inversion pulse and the k‐space center of the slice). The slice order changes after each inversion pulse to acquire enough sampling points along the longitudinal recovery curve for each slice. T_1_ maps can be calculated using a 2‐parameter fit model (assumes perfect inversion pulse efficiency) or 3‐parameter fit model (assumes imperfect inversion pulse efficiency). Although differences in myocardial T_1_ times can be evidence of cardiomyopathies, they also can be caused by myocardial fat infiltration.^14^

In addition to the valuable clinical information provided by myocardial T_1_ maps, quantification of the epicardial fat volume can play a significant role in evaluating cardiovascular risk.^15^ Epicardial fat has been shown to be associated with various cardiovascular diseases such as atrial fibrillation and coronary atherosclerosis.^16^, ^17^ Assessment of epicardial fat volume is usually achieved with Dixon‐based sequences, which can provide water‐only and fat‐only images simultaneously.^18^, ^19^, ^20^

Adding both T_1_ mapping and fat quantification to a cardiovascular MRI protocol unnecessarily prolongs overall scan time. The goal of this study was therefore to develop and evaluate a novel imaging sequence for joint T_1_ mapping and fat/water to allow simultaneous quantification of myocardial fat and T_1_ with no penalty in imaging time. Phantom and in vivo experiments were performed to compare the measured T_1_ between the STONE and joint T_1_‐fat/water separation sequence. In vivo epicardial fat volume were measured and compared with Dixon and joint T_1_‐fat/water separation sequence.

## 1 METHODS

### 1.1 Proposed sequence scheme

#### 1.1.1 Acquisition

**Figure 1:**
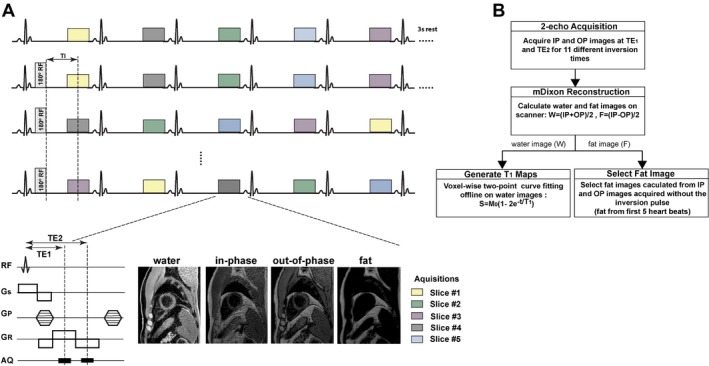
A, Pulse sequence diagram of the proposed joint T_1_‐fat/water separation sequence. For each heartbeat, 2 images were acquired (in phase and out of phase). For the first 5 heartbeats, images are acquired without inversion pulse. After a 3‐second rest period, a 180 ° inversion pulse was applied and data acquired in the subsequent 5 heartbeats with the first inversion time (TI_1_). This is then repeated 5 times with different slice orders. The same sequence block is repeated 1 more time with second inversion time (TI_2_). In total, the sequence results in 220 images. B, Postprocessing steps for the joint T_1_‐fat/water separation sequence for T_1_ mapping and fat quantification. IP, in phase; OP, out of phase

The schematic of the proposed imaging sequence is shown in Figure 1A, which combines the STONE sequence using a gradient‐echo (GRE) imaging readout with a Dixon water/fat separation technique. The standard STONE technique is used to obtain a T_1_ map of 5 slices simultaneously. At each heartbeat, 2 single‐shot images are acquired with different TEs: the first image with fat and water signal in phase and the second one with fat and water signal out of phase. With the proposed sequence, the first in‐phase and out‐of‐phase images are acquired without the inversion pulse for each slice followed by a 3‐second pause cycle for magnetization recovery. Subsequently, in‐phase and out‐of‐phase images are acquired after applying a nonselective inversion pulse and the inversion time TI_1_ as defined as the time interval between the center of the inversion pulse and the center of the k‐space. The inversion pulse and the 5 acquisitions are then repeated 5 times with different slice order and by keeping the distance between 2 adjacent slices maximal. Subsequently, the same data acquisition is repeated with an inversion time TI_2_. The sequence was performed during free breathing with a pencil beam navigator positioned on the right hemidiaphragm, allowing for prospective respiratory motion tracking. To reduce in‐plane motion between the T_1_‐weighted images of each slice, a nonrigid image registration was applied.^21^, ^22^

#### 1.1.2 Reconstruction

To calculate water and fat images, mDixon fat/water separation reconstruction was performed using the scanner's inline processing software.^19^ The proposed sequence yields 44 images for each slice corresponding to inversion times of ∞, TI, TI+1RR, TI+2RR, TI+3RR, and TI+4RR (RR being the duration of 1 heart cycle), which results in 220 reconstructed images in total. A 2‐parameter exponential fitting model was used offline using in‐house software written in MATLAB (The MathWorks Inc., Natick, MA) to calculate the T_1_ map from 55 water‐only images, $S=M_{0}\left(1-2e^{\frac{-t}{T_{1}}}\right)$, in which M_0_ is the full longitudinal magnetization, *t* is the inversion time, and T_1_ is the longitudinal relaxation time (Figure 1B).

### 1.2 Phantom

Two phantom experiments were performed to test and validate the proposed method. All scans were performed on a 1.5T scanner (Philips Achieva, Best, the Netherlands) equipped with a 32‐element cardiac coil to evaluate the proposed sequence.

#### 1.2.1 Experiment I

The purpose of the first phantom experiment was to assess the proposed sequence performance in terms of correctly estimating the T_1_ values and separating the fat from the water signals. A phantom containing fat‐free vials with NICl_2_‐doped agarose gel and T_1_ values between 300 and 1450 ms and T_2_ values between 50 and 200 ms was used. A bottle of oil was placed adjacent to the agarose phantom to represent fatty tissues. Image acquisition parameters were as follows: TR/TE = 7.1/4.6 ms for the STONE sequence, TR/TE1/TE2 = 7.9/2.3/5.4 ms for the joint T_1_‐fat/water separation sequence, TI = ∞, 109, 1109, 2109, 3109, 4109, 350, 1350, 2350, 3350, and 4350 ms, flip angle = 10°, in‐plane resolution = 2 × 2 mm^2^, slice thickness = 8 mm, FOV = 180 × 180 mm^2^, SENSE factor = 2, partial Fourier factor = 0.75, turbo‐field‐echo factor = 56, and 10 linear ramp‐up pulses. The electrocardiogram signal was simulated for 60 bpm.

As a reference, an inversion‐recovery spin‐echo sequence was used. Sixteen images were acquired with different TIs (50, 100, 200, 300, 400, 500, 600, 700, 800, 900, 1000, 1250, 1500, 1750, 2000, and 3000 ms). The imaging parameters were as follows: TR = 10 seconds, TE = 10 ms, FOV = 200 × 220 mm^2^, flip angle = 90°, voxel size = 1.2 × 1.2 mm^2^ and slice thickness = 8 mm.

#### 1.2.2 Experiment II

A second phantom experiment was performed using the same parameters to demonstrate the feasibility of the proposed sequence to estimate T_1_ in voxels containing both water and fat. To investigate the effect of the number of echoes of the T_1_‐fat/water separation technique, the same phantom was scanned with 3 echoes. A dedicated phantom was designed for this experiment. The phantom consisted of 4 vials with different concentrations of mayonnaise (i.e., fat) mixed with gadolinium (Gd)‐doped water (10, 20, 30, and 40 mL of mayonnaise added to 40, 30, 20, and 10 mL of the water‐Gd mixture). The 4 vials were then immersed in the Gd‐doped water container. The mayonnaise consisted of egg yolks, oil, and vinegar (10 g of fat and 1.5 g of saturated fat per serving).

### 1.3 In vivo

Experiments were HIPPAA compliant and approved by our Institutional Review Board. Informed consent was obtained from all individual participants.

To evaluate the proposed sequence, we prospectively recruited 8 healthy adult subjects (24 ± 5 years, 3 males) and 9 patients (60 ± 9 years, 8 males). Patients had known atrial fibrillation (5 patients), nonischemic cardiomyopathy (3 patients), and left ventricular hypertrophy (1 patient). Each subject was scanned using 3 different sequences: (1) STONE T_1_ mapping, (2) joint T_1_‐fat/water separation sequence, and (3) Dixon alone. Imaging parameters were as follows: TR/TE = 7.1/4.6 ms for STONE, TR/TE1/TE2 = 7.5/2.2/5.2 ms for joint T_1_‐fat/water separation sequence, TI = ∞, 109, 1109, 2109, 3109, 4109, 350, 1350, 2350, 3350, and 4350 ms, flip angle = 10°, in‐plane resolution = 2 × 2 mm^2^, slice thickness = 8 mm, FOV = 300 × 300 mm^2^, SENSE factor = 2, partial Fourier factor = 0.75, turbo‐field‐echo factor = 56, and 10 linear ramp‐up pulses. The average heart rate was 65 ± 7 bpm. Images were acquired during free breathing and the nominal scan time was 1 minute 35 seconds for a heart rate of 60 bpm. The fat volume measured from 5 slices corresponding to STONE T_1_ map locations were used as the “true” fat volume. The imaging parameters for the Dixon sequence were as follows: TR = 4.9 ms, TE1/TE2 = 1.52/3.3ms, FOV = 300 × 300 mm^2^, flip angle = 15°, voxel size = 2 × 2 mm^2^, and slice thickness = 8 mm. An inline mDixon water/fat separation reconstruction was used to provide water and fat images.

### 1.4 Ex vivo human heart

To further evaluate the performance of our sequence, an ex vivo human heart scan was performed with STONE, joint T_1_‐fat/water separation sequence, Dixon, and inversion‐recovery spin‐echo sequence. We explanted a human heart from a 66‐year‐old male patient who died due to complications from cardiovascular disease. The patient had a mixed cardiomyopathy disease with prior myocardial infarction and significant fatty infiltration of the left and right ventricular myocardium and septum, predominantly subepicardial. Microscopic evaluation performed in a limited autopsy showed subepicardial and midmyocardial replacement fibrosis with associated fatty infiltration. The imaging parameters were the same as for the phantom scans with STONE, joint T_1_‐fat/water separation, Dixon, and the spin‐echo sequences.

### 1.5 Analysis

Images were transferred to a separate workstation for analysis. For the phantom data, a region of interest was manually drawn on each vial in both STONE and joint T_1_‐fat/water separation T_1_ maps and the reported T_1_ value was the mean T_1_ of the respective region of interest in each vial. For the in vivo experiments, the myocardium was divided into 16 segments according to the American Heart Association myocardial segment model.^23^ Epicardial and endocardial contours were defined manually for each T_1_ map in all 5 slices. To measure the fat area, the region of interest was drawn manually to contour the epicardial border using OsiriX software version 7.5.1 in all 5 slices of the fat‐only images. Precision of native T_1_ mapping was evaluated using a myocardial segment‐based analysis and a subject‐based analysis. The precision was defined as the average of the SD of the T_1_ values, and the coefficient of variation represents the ratio of the SD to the mean. The fat volume was calculated by ($\sum^{}{\text{fat}}_{\text{area}}$) × slice thickness.

Variables are expressed as mean ± SD and compared using an unpaired Student's t‐test or Mann‐Whitney nonparametric test if not normally distributed. A *P* value of less than .05 was considered statistically significant. The intraclass correlation coefficient was assessed for both T_1_ measurements and fat volume. The Pearson correlation coefficient was used to examine the relationship between the STONE and T_1_‐fat/water separation technique. All analyses were performed using the SPSS (version 19.0, International Business Machines Inc, Chicago, IL).

## 2 RESULTS

### 2.1 Phantom study

**Figure 2:**
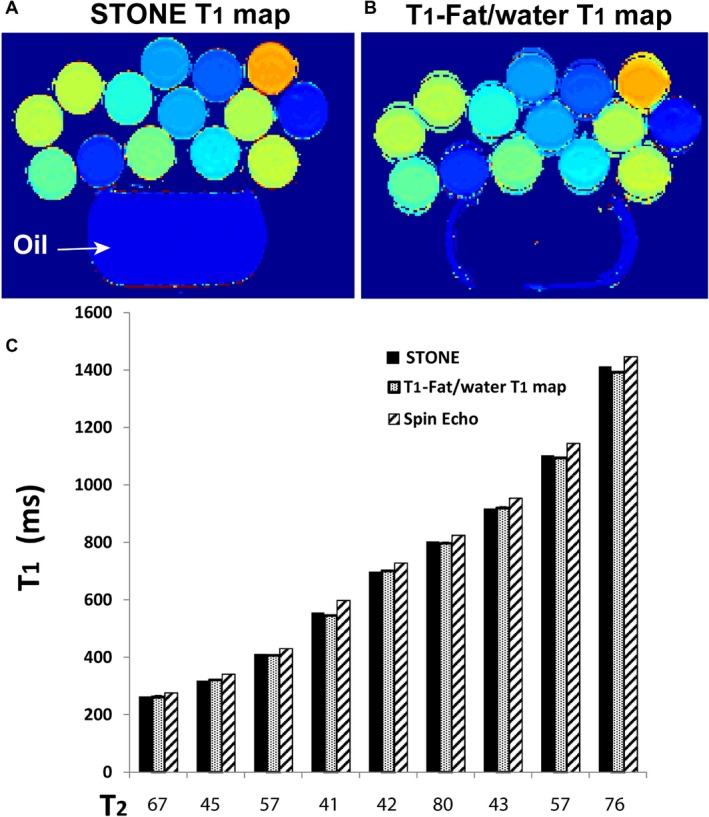
Phantom images acquired with the slice‐interleaved T_1_ (STONE) (A) and joint T_1_‐fat/water separation (B) techniques of vials with different T_1_ and T_2_ values and a bottle of canola oil (14 g of fat in 1 teaspoon) (top). C, Measured T_1_ values of the different vials acquired with the 2 sequences and reference inversion‐recovery spin‐echo sequence. The x‐axis indicates T_2_ values measured with a reference multi‐echo spin‐echo‐based sequence for each vial

**Figure 3:**
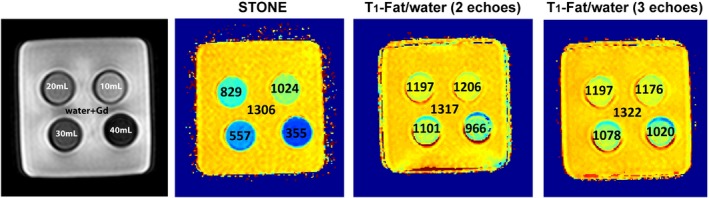
Phantom images acquired with the STONE and joint T_1_‐fat/water separation sequences (2 echoes and 3 echoes) demonstrate the feasibility of accurate T_1_ mapping with the proposed joint T_1_‐fat/water separation even in a voxel with a mixture of water and fat. The phantom consists of vials that contain a mixture of mayonnaise (fat) and gadolinium (Gd)‐doped water. The mayonnaise‐to‐water proportion in each vial was varied to imitate different ratios of fat infiltration. This was done by maintaining the amount of Gd‐doped water (50 mL) and by adding different amounts of mayonnaise (10 mL, 20 mL, 30 mL, and 40 mL). The calculated T_1_ values with STONE decreased with an increasing amount of mayonnaise (1024 ms, 829 ms, 557 ms, and 355 ms) but did not change with the joint T_1_‐fat/water separation sequence with 2 echoes (1206 ms, 1197 ms, 1101 ms, and 966 ms) and 3 echoes (1176 ms, 1197 ms, 1078 ms, and 1020 ms)

T_1_ maps of the phantom and oil vials that were acquired with the STONE and the joint T_1_‐fat/water separation sequence are shown in Figure 2. The T_1_ maps were generated using the 2‐parameter fit model applied to the STONE and water‐only images. The bar graph (Figure 3C) shows the measured T_1_ times for each vial. There was no difference between the T_1_ values measured with the STONE and the joint T_1_‐fat/water separation sequences (*P* = .1).

T_1_ maps of the vials with different concentration of fat are shown in Figure 3. The calculated T_1_ by the STONE sequence (1024 ms, 829 ms, 557 ms, and 355 ms) and by the joint T_1_‐fat/water separation sequence with 2 echoes (1206 ms, 1197 ms, 1101 ms, and 966 ms) and 3 echoes (1176 ms, 1197 ms, 1078 ms, and 1020 ms) for water doped with Gd and 10 mL, 20 mL, 30 mL, and 40 mL of mayonnaise, respectively. differed significantly. By increasing the concentration of the mayonnaise, the T_1_ values with the STONE sequence decreased (*P* = .12). The percent difference of the measured T_1_ between the reference (water doped with Gd [0% mayonnaise]) and 20%, 30%, 50%, and 80% mayonnaise water/Gd mixture by STONE was 22%, 36%, 57% and 73%, whereas the difference was significantly lower with the joint T_1_‐fat/water separation sequence with 2 echoes (8%, 9%, 16%, and 26%) and 3 echoes (11%, 9%, 18%, and 23%). There was no statistically significant difference between T_1_ measured with 2 echoes and 3 echoes (*P* = .9). Therefore, only 2 echoes were used for all experiments in this study.

### 2.2 In vivo

**Figure 4:**
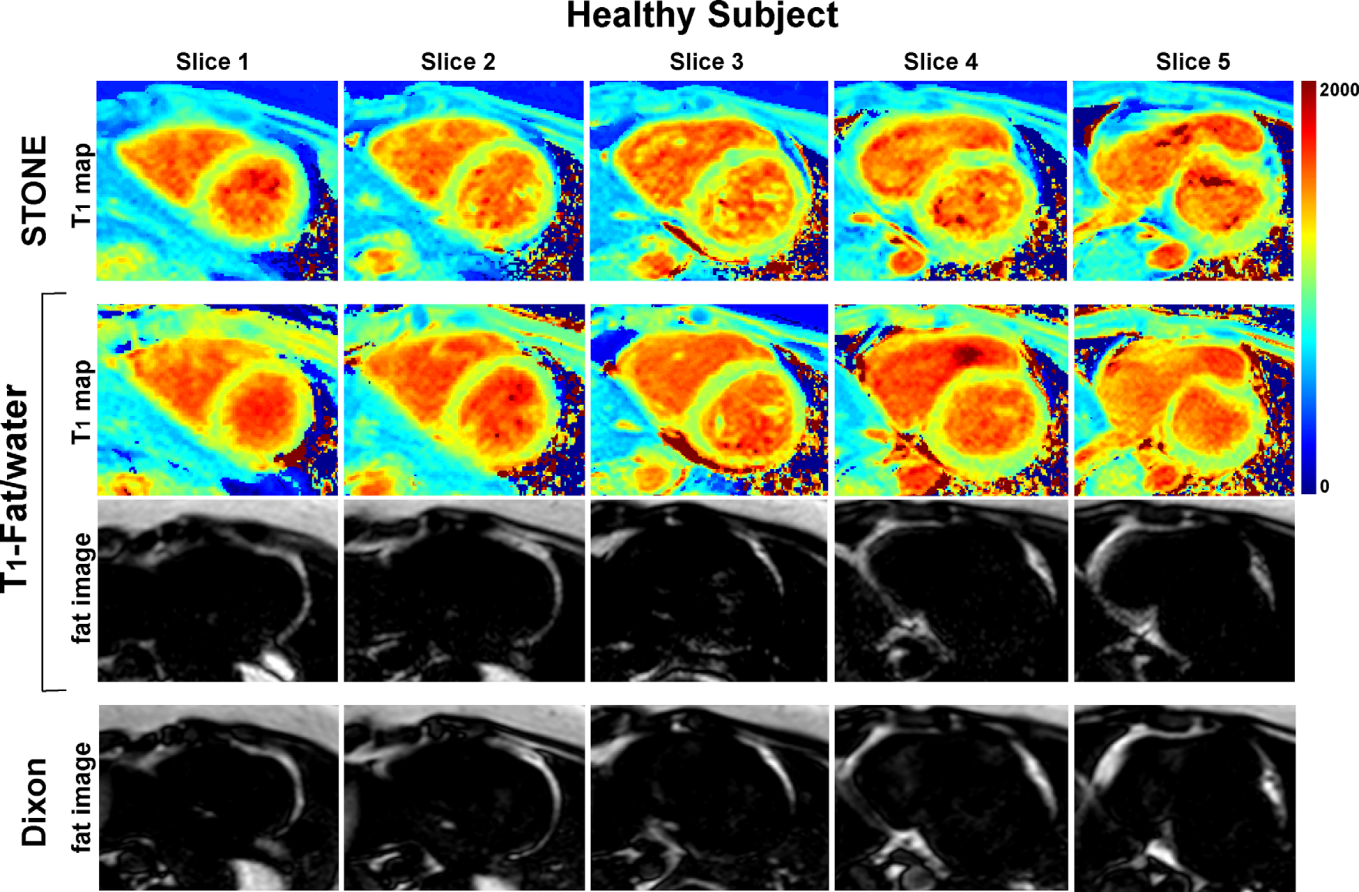
Representative T_1_ maps and fat images of a healthy subject acquired with the STONE (top row), T_1_‐fat/water (middle 2 rows), and Dixon (bottom row) sequences

Representative in vivo T_1_ maps with STONE and joint T_1_‐fat/water separation sequences in a healthy subject and a patient are shown in Figure 4 and Supporting Information Figure S1, respectively. The T_1_ maps with the proposed sequence visually appear homogeneous across all slices with no visible artefacts. In the 17 subjects examined, the average T_1_ value measured with STONE was 1067 ± 18 ms and 1077 ± 24 ms (*P* = .6) with the joint T_1_‐fat/water separation sequence. There is a trend of higher precision with the joint T_1_‐fat/water separation sequence compared with STONE in a subject‐based analysis (STONE: 71 ± 10 ms, T_1_‐fat/water separation: 65 ± 10 ms, *P* = .06) and significantly higher precision in a segment‐based analysis (STONE: 64 ± 20 ms, T_1_‐fat/water separation: 55 ± 15 ms, *P* < .01), due to the higher SNR of the joint T_1_‐fat/water separation sequence. The coefficient of variation was 2.9% and 2.4% in subject‐based analysis, and 2.1% and 2.0% in segment‐based analysis for STONE and T_1_‐fat/water separation, respectively. There was no significant difference in the epicardial fat volume between the joint T_1_‐fat/water separation and the conventional Dixon sequence (54.3 ± 33 cm^3^ versus 52.4 ± 32 cm^3^, *P* = .8). The intraclass correlation coefficients of subject‐based T_1_ measurements and epicardial fat volume were 0.92 (95% confidence interval [CI]: 0.8‐0.97) and 0.99 (95% CI: 0.92‐0.99). There were excellent correlations for the measured T_1_ values (r = 0.99, *P* < .001) and fat volume (r = 0.92, *P* < .001) between the STONE and T_1_‐fat/water separation technique.

The acquisition time was 1 minute 35 seconds for both STONE and joint T_1_‐fat/water sequences and 35 seconds for the Dixon‐only method for a heart rate of 60 bpm.

There was no significant difference in native myocardial T_1_ in healthy subjects and patients between the 2 sequences. The average T_1_ value was 1069 ± 18 ms and 1076 ± 26 ms (*P* = .5) in healthy subjects and 1071 ± 17 ms and 1075 ± 23 ms (*P* = .9) in patients measured with STONE and T_1_‐fat/water separation sequences. However, there was a difference with regard to precision in both patients and healthy subjects between STONE and T_1_‐fat/water separation sequences. The average segment‐based precision was 61 ± 16 ms and 53 ± 14 ms (*P* = .07) in healthy subjects and 67 ± 23 ms and 57 ± 16 ms (*P* < .001) in patients measured with STONE and T_1_‐fat/water separation sequences. The average subject‐based precision was 66 ± 9 and 59 ± 6 ms (*P* = .04) in healthy subjects and 73 ± 8 ms and 67 ± 12 ms (*P* = .5) in patients measured with STONE and T_1_‐fat/water separation sequences. The intraclass correlation coefficients of subject‐based T_1_ measurements for patients and healthy subjects were 0.92 (95% CI: 0.65‐0.98) and 0.93 (95% CI: 0.73‐0.98), respectively. The intraclass correlation coefficients of epicardial fat volume for patients and healthy subjects were 0.96 (95% CI: 0.83‐0.99) and 0.98 (95% CI: 0.94‐0.99), respectively.

### 2.3 Ex vivo human heart

**Figure 5:**
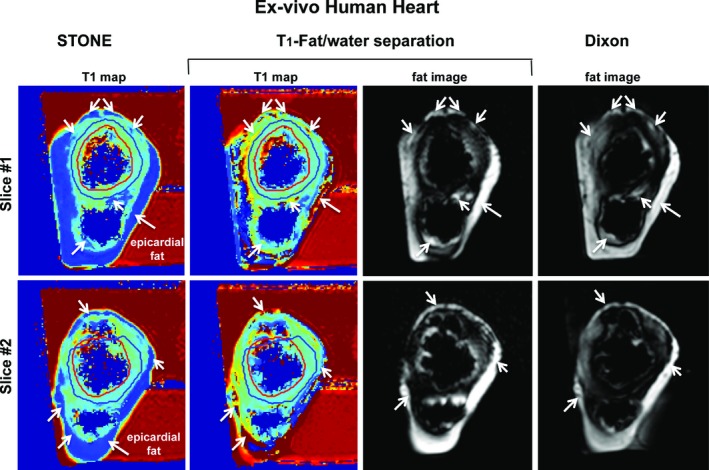
T_1_ maps and fat images of an ex vivo human heart with the STONE (first column), T_1_‐fat/water (middle 2 columns), and Dixon (last column) sequences. The measured T_1_ values in the ex vivo human heart were 731 ± 150 ms with STONE, 800 ± 44 ms with T_1_‐fat/water separation, and 918 ± 49 ms with the inversion‐recovery spin‐echo sequence. The epicardial fat volumes were 110 cm^3^ with Dixon and 106 cm^3^ with joint T_1_‐fat/water separation sequence. The left ventricular endocardial (red line) and epicardial (blue) were manually drawn on T_1_ maps, carefully excluding the areas affected by image artifacts. White arrows indicate areas of the subepicardial fat

Figure 5 shows example slices from the ex vivo human heart. The left ventricular endocardial and epicardial borders were drawn manually on myocardial slices of the T_1_ maps by carefully excluding areas affected by image artifact. Subsequently, these regions of interest were copied to corresponding images. The measured T_1_ values in the ex vivo human heart were 731 ± 150 ms, 800 ± 44 ms, and 918 ± 49 ms for the STONE, T_1_‐fat/water separation, and inversion‐recovery spin‐echo sequences, respectively. The percent difference of the measured T_1_ between the spin echo and T_1_‐fat/water separation (12%) was lower than that of the STONE (20%) sequence. With the proposed sequence the measured T_1_ is closer to the spin‐echo value. Because of significant fatty infiltration of the left ventricle myocardium, we were not able to identify areas in the myocardium where “true” T_1_ of the myocardium can be measured, which may explain shorter native myocardial T_1_ at 1.5 T. The epicardial fat volumes were 110 cm^3^ and 106 cm^3^ with the Dixon and joint T_1_‐fat/water separation sequences, respectively.

## 3 DISCUSSION

In this study, a joint T_1_‐fat/water separation sequence was developed, which separates the water and fat images and calculates T_1_ maps based only on the water content of the tissues. The joint T_1_‐fat/water separation sequence was applied successfully to phantom, healthy subjects and patients, and provided visually homogeneous T_1_ maps. In addition, fat volume quantified in vivo with the joint T_1_‐fat/water separation sequence provided similar values as with the conventional Dixon fat/water separation technique.

The main benefits of the proposed sequence are (1) shortening the scan time by combining T_1_ mapping and fat quantification with the joint T_1_‐fat/water separation sequence, (2) higher precision compared with the conventional T_1_ mapping sequence, and (3) fat images and T_1_ maps are spatially matched as reconstructed from the same data set. Therefore, direct comparison of the calculated T_1_ maps and fat images is facilitated without the need for further image registration.

In this study, 2 echoes were used. Increasing the number of echoes for the Dixon reconstruction can improve the B_0_ inhomogeneity correction and generate true fat and water separation, which is important especially for quantification of fat fraction. However, 3‐point Dixon methods would have restricted the ability to optimize spatial resolution and are more susceptible to inconsistencies in the phase error and most importantly increase the total scan time.^17^ Because the fat image in this study was used to calculate fat volume only, not fat fraction, the 2‐point Dixon acquisition used here was deemed sufficient.

Because fat has a short T_1_ value, it can influence the measured myocardial T_1_ values and consequently lead to lower or higher T_1_ relaxation times depending on the T_1_ mapping sequence.^24^ Intramyocardial fat is correlated with various cardiomyopathies but is also present to some degree in healthy subjects.^25^, ^26^ Intramyocardial fat has been observed primarily in patients with myocardial infarction,^27^, ^28^ cardiac lipoma,^29^ dilated cardiomyopathy,^30^ and arrhythmogenic right ventricular dysplasia.^31^, ^32^ In normal myocardium, it has frequently been seen in the right ventricle (16%‐43%) 33, 34 and can increase with age. Small quantities of fat have been observed in the left ventricle.

To demonstrate that the proposed sequence can accurately quantify T_1_ in a voxel with different percentage of fat and water, a mayonnaise phantom experiment was performed. With the joint T_1_‐fat/water separation sequence, the measured T_1_ was slightly lower in the vials with increasing percentage of mayonnaise compared with that measured with the Gd‐doped water. This observation may be explained by the fact that mayonnaise is not 100% fat; it is a stable emulsion of oil, egg yolk, vinegar, and various spices and thus may change the T_1_ of water. As the percentage of egg yolk with vinegar may have slightly changed by changing the fat content, this by itself may have led to different T_1_ relaxation times.

Balanced SSFP is often used in T_1_ mapping sequences such as MOLLI.^4^ However, we combined the STONE T_1_ mapping sequence with GRE readout because the Dixon water/fat separation is typically performed with a multi‐echo GRE imaging. Despite lower SNR of the GRE imaging readout compared with SSFP, STONE with GRE provides similar T_1_ values, precision, and reproducibility compared with SSFP at 1.5 T.^35^

This study has several limitations. We did not investigate the performance of the proposed sequence for postcontrast T_1_ mapping. Furthermore, to compare the accuracy of fat quantification, the manual tracing of epicardial fat was performed, which can be challenging due to poor visualization of the pericardium. Magnetic resonance spectroscopy may be required to validate the response of the measured T_1_ by the joint T_1_‐fat/water separation sequence for the various percentages of intramyocardial fat. However, we did not measure the intramyocardial fat content, as it is challenging, and it was not the primary aim of this project.

## 4 CONCLUSIONS

We devised and evaluated a joint water/fat T_1_ mapping sequence that allows simultaneous quantification of epicardial fat content and myocardial T_1_ relaxation time without increasing the overall scan time. The sequence was in good agreement with the STONE T_1_ mapping technique and a 2‐point Dixon water/fat separation technique.

## Supporting information

Additional Supporting Information may be found in the supporting information tab for this article.

**FIGURE S1** Representative T_1_ maps and fat images of a patient acquired with the STONE (top row), T_1_‐fat/water (middle 2 rows), and Dixon (bottom row) sequences.
