# Supplementary material for: Imaging sequence for joint myocardial T 1 mapping and fat/water separation
Source: Magn Reson Med. 2018 Jul 29;81(1):486–94. doi: 10.1002/mrm.27390 (PMC6258274; doi:10.1002/mrm.27390)
Supplement: Supplementary file 1 — FIGURE S1 Representative T1 maps and fat images of a patient acquired with the STONE (top row), T1‐fat/water (middle 2 rows), and Dixon (bottom row) sequences. [file MRM-81-486-s001.pdf]

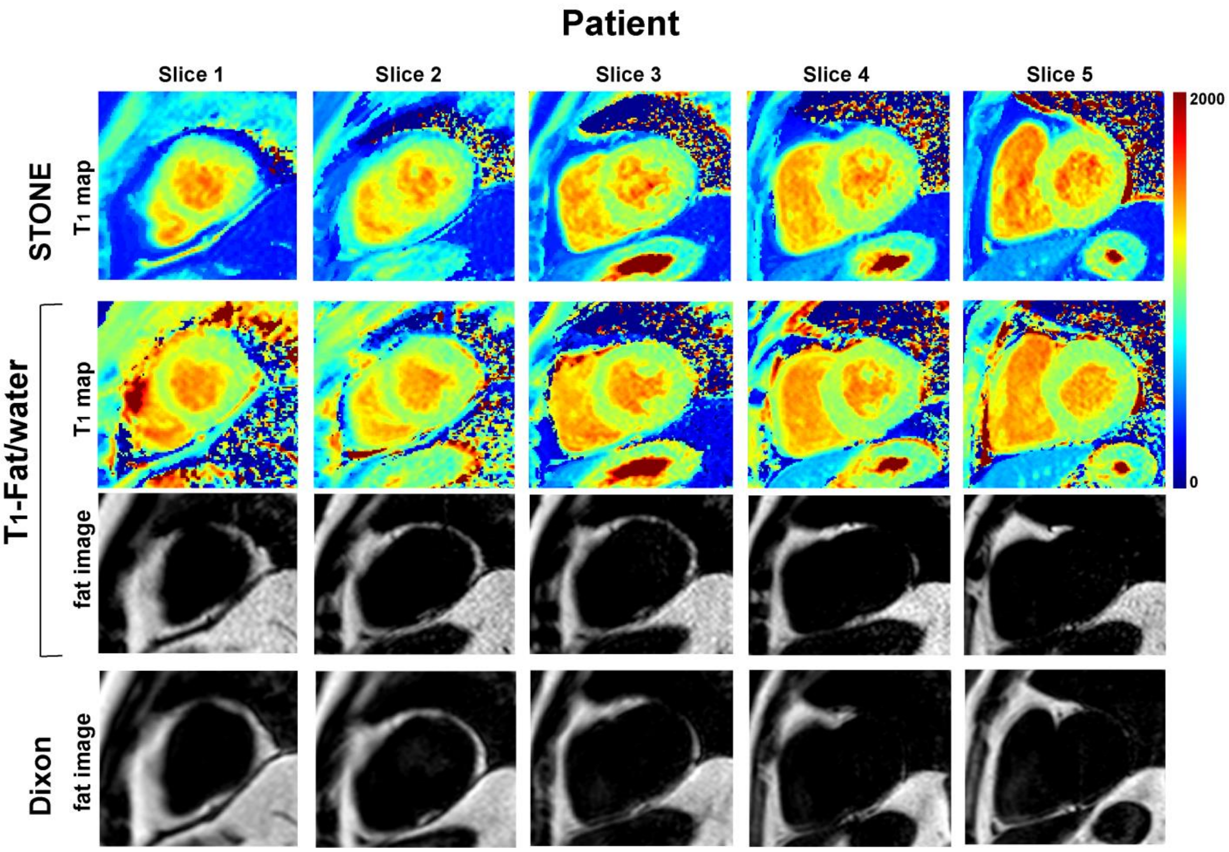

Figure S1: Representative T1 maps and fat images of a patient acquired with STONE (top row), T1-fat/water (middle two rows) and Dixon (bottom row) sequence.
